# The Stance Leads the Dance: The Emergence of Role in a Joint Supra-Postural Task

**DOI:** 10.3389/fpsyg.2017.00718

**Published:** 2017-05-09

**Authors:** Tehran J. Davis, Gabriela B. Pinto, Adam W. Kiefer

**Affiliations:** ^1^Center for the Ecological Study of Perception and Action, Department of Psychological Sciences, University of Connecticut, StorrsCT, USA; ^2^CAPES Foundation, Ministry of Education of BrazilBrasília, Brazil; ^3^Division of Sports Medicine, Cincinnati Children’s Hospital Medical Center, CincinnatiOH, USA; ^4^Department of Pediatrics, College of Medicine, University of Cincinnati, CincinnatiOH, USA; ^5^Center for Cognition, Action and Perception, Department of Psychology, University of Cincinnati, CincinnatiOH, USA

**Keywords:** interpersonal coordination, joint action, movement dynamics, recurrence quantification analyses, self-organization

## Abstract

Successfully meeting a shared goal usually requires co-actors to adopt complementary roles. However, in many cases, who adopts what role is not explicitly predetermined, but instead emerges as a consequence of the differences in the individual abilities and constraints imposed upon each actor. Perhaps the most basic of roles are *leader* and *follower*. Here, we investigated the emergence of “leader-follower” dynamics in inter-personal coordination using a joint supra-postural task paradigm (Ramenzoni et al., 2011; Athreya et al., 2014). Pairs of actors were tasked with holding two objects in alignment (each actor manually controlled one of the objects) as they faced different demands for stance (stable vs. difficult) and control (which actor controlled the larger or smaller object). Our results indicate that when actors were in identical stances, neither led the inter-personal (between actors) coordination by any systematic fashion. Alternatively, when asymmetries in postural demands were introduced, the actor with the more difficult stance led the coordination (as determined using cross-recurrence quantification analysis). Moreover, changes in individual stance difficulty resulted in similar changes in the structure of both intra-personal (individual) and inter-personal (dyadic) coordination, suggesting a scale invariance of the task dynamics. Implications for the study of interpersonal coordination are discussed.

## Introduction

Two friends passing a cup of coffee involves the coordination of no fewer than 2 arms, 8 joints, and 50 muscles spread across two separate bodies. To avoid a mishap, each person must, at minimum, continuously track the positions and orientation of one another’s hands, and act so as to mutually align their movements within a very narrow window of space and time. Indeed, these sorts of exacting perceptual and motor demands are necessary in even the most basic of joint tasks. And yet (perhaps quite remarkably) waiters frequently pass plates, workers routinely pass tools, and children successfully pass toys with little thought or concerted effort.

It is argued that successful joint actions, such as passing a cup, result from the formation of softly assembled, coordinative structures between multiple actors (Black et al., 2007; Shockley et al., 2009; Riley et al., 2011; Saltzman and Caplan, 2015). A central theme of this framework is the appeal to principles of emergent self-organization when explaining how a given number of degrees of freedom (e.g., joints and neuromuscular groups) might become functionally coordinated. For individual actors, “solving” this problem involves the recruitment or reduction of these degrees of freedom in accord with the constraints placed upon the system during the execution of action (Bernstein, 1967; Gelfand and Tsetlin, 1971; Turvey, 2007). According to the inter-personal synergy hypothesis (Riley et al., 2011), the coordination of joint actions between individuals is the result of similar processes—mutual constraint and synergistic organization across two or more people’s bodily and cognitive states.

Some support for this position comes by way of research investigating the organization of body segments (e.g., hand and torso) when two people engage in a joint supra-postural precision task (a task where two standing co-actors must make very precise and exacting movements while maintaining upright balance). It has been argued that successes in these sorts of tasks are built upon a nested hierarchy of intra-personal and inter-personal coordinative structures between hand and postural control (Riley et al., 2011; Ramenzoni et al., 2012) that emerge to meet and continuously adapt to the evolving task demands. For example, when a single actor performs a precision grasping or aiming task, the activity of separate body segments within the actor show signs of mutual interdependence: activity from other body segments, postural control systems and even respiration (Balasubramaniam et al., 2000; Kuznetsov et al., 2011; Ramenzoni et al., 2012) act in a compensatory manner to facilitate the actor’s goal.

Ramenzoni et al. (2011) found evidence that analogous synergistic processes occur across individuals that are cooperating to complete a shared task. In their experiment, one member of a dyad held a pointer-like object while a partner held a ring-like object. Dyads were then tasked with manually aligning their respective objects so that the pointer remained within the perimeter of the ring (without touching) for the duration of each trial. The task’s difficulty was manipulated at the inter-personal level by varying the diameter of the ring—smaller rings placed more exacting precision demands between the actors. Intra-personal task difficulty was manipulated by independently changing the stance of each actor: Actors either stood with a normal base of support (their feet shoulder-width apart) or stood in a heel-to-toe tandem stance that narrowed the base of support and required more effort to maintain upright balance. Both challenges were typically met with increases in the degree and the stability of coordination within and between actors. For example, decreases in ring size corresponded to increases in the amount of shared activity between actors’ hands and postures as well as increases in intra-personal hand-posture coordination (as measured by cross-recurrence quantification analysis (CRQA), see Section “CRQA: Global Dynamics and Leader-Follower Analyses”). While inter-personal coordination between actors’ hands was compromised (but not eliminated) in conditions when one or both actors were in the tandem stance condition, a compensatory increase in the coordination of co-actor’s postural activity was observed.

More recently, Athreya et al. (2014) demonstrated that comparable patterns of coordination emerge even in instances where information about the movements of co-actors is limited. Pairs of participants were asked to perform a similar manual alignment task, this time aligning two laser pointers on a black screen. Participants performed this task under conditions where they could see their partner as well as conditions were their partner remained unseen. In the unseen condition, participants still had task-specific information about their confederate via the movement and position of the confederate’s laser. Athreya et al. (2014) observed that measures of inter-personal hand coordination remained consistent across both conditions. Importantly, inter-personal torso coordination in the unseen condition was still present (though reduced compared to the seen condition). This result suggests that the postural coordination observed in this task was not entirely an incidental product of visual entrainment (Varlet et al., 2011), but instead may have been closely tied to the detection of information related to the individual and shared task demands.

The two aforementioned investigations questioned how manipulating task demands lead to changes in the coordination dynamics within and across actors. In the present study, we asked an equally important question—how do differences in individual demands result in the spontaneous organization of distinct roles between actors? It is rarely the case that individuals performing a shared task mirror one another’s actions. Instead, many joint tasks demand that individuals perform complimentary actions, or adopt roles, to reach their desired ends. For example, “Having a conversation” implies a “speaker” and a “listener” and “Passing a ball” requires a “thrower” and “catcher.” More generally, during joint tasks there are actors that lead or initiate an interaction (e.g., speakers and throwers), and actors that follow (listeners and catchers). In many cases these leader-follower roles are not defined *a priori*, but instead emerge spontaneously provided asymmetries in the individual abilities, constraints, and goals of each actor. In Ramenzoni et al.’s (2011) study, such task asymmetries were present in each individuals’ manual and postural demands. Actors were either “rings” setting the boundary constraints of the joint task, or they were “pointers” tasked with maintaining their position within the bounds of the ring. At the same time, the actors faced different challenges to stance—actors standing with their feet in tandem stance encountered greater difficulty maintaining postural stability than actors with their feet shoulder-width apart.

Recently, Bosga et al. (2010) proposed that a framework developed for describing the coordination between multiple joints may be useful in understanding leader-follower dynamics between multiple agents (or multiple body segments across actors). The leading joint hypothesis (LJH) (Dounskaia, 2005, 2010) suggests that individual joints in a multi-joint action play different roles in the production of the global movement, where the leading joint acts as a linchpin for the organization of movement for the remaining joints. Typically, the leading joint emerges out of the interplay of task constraints and the functional and bio-mechanical linkages between body segments. During a multi-articular movement, mechanical interactions between interdependent body segments produce varying amounts of torque at each joint. The leading joint, typically the joint with a mechanical advantage, exerts additional movement torques on the subordinate joints. This results in greater movement variability and increased complexity in movements around the subordinate joints while maintaining relatively low variability and complexity of movements around the leading joint.

Bosga et al. (2010) demonstrated that analogs to the “leading-joint” may be found in multi-agent rhythmic coordination. They tasked pairs of actors with cooperatively moving a rocking board side-to-side, while measuring the enclosed angles of various joints about the actors’ bodies. Kinematic analysis focused on the angular displacements and continuous relative phase angles of actors’ joints, and leader-follower relationships were determined using time-lagged cross-correlations of these values. Analysis of the inter-personal coordination dynamics often revealed the presence of a leading actor whose movement kinematics were consistent with those predicted by the LJH—namely measures of angular displacement variability about the joints of leading rockers tended to be lower than variability about the joints of followers.

With this in mind, we had two specific aims for this study. First, we investigated how patterns of intra-personal and inter-personal coordination changed as a function of each individual’s stance demands and disk control. At the intra-personal level, we expected that increases in stance difficulty would result in increases in movement variability about the torso of individual actors. More, we expected that these increases would be met with increases in the regularity (Paterno et al., 2015), complexity and intermittency (associated with functional flexibility, Kiefer and Myer, 2015) of intra-personal coordination between these two body segments. Such changes would reflect the reorganization of the available degrees of freedom to insulate the hand from the effects of increased torso variability. At the level of inter-personal coordination, we predicted similar patterns of effects—that increases in shared-stance difficulty would result in increases in the regularity, complexity, and intermittency of coordination between the two actors as they encounter greater difficulties to completing the task.

Our second, more central aim was to investigate the degree to which asymmetries in individual task demands corresponded to the emergence of leader-follower dynamics in inter-personal coordination. For example, consistent with the LJH, we predicted that the member of the dyad whose body segments exhibited greater movement variability would have an increased likelihood as acting as a “subordinate joint” or follower in the joint task. Also, given that the LJH makes claims regarding complexity, we were inclined to predict that the actor showing greater complexity in intra-personal coordination would likely emerge as a follower in the task. Given our above predictions about the relative effects of stance difficulty, this leader-follow dynamic was expected to be most pronounced in conditions when the actors faced asymmetrical stance demands. That is, we predicted that actors facing less challenges to upright stance would tend to lead the coordination when their partners were in the more difficult tandem stance. When both actors faced similar stance demands, the regularity and systematic nature of this leader-follower relationship were expected to be reduced.

## Materials and Methods

### Participants

We recruited 24 undergraduates (14 women and 10 men) in pairs (12). All participants reported being free of recent injury and had normal or corrected to normal vision. Informed consent was obtained in agreement with the University of Connecticut Institutional Review Board’s standards and practices. Undergraduates received course credit for their participation.

### Apparatus

**FIGURE 1:**
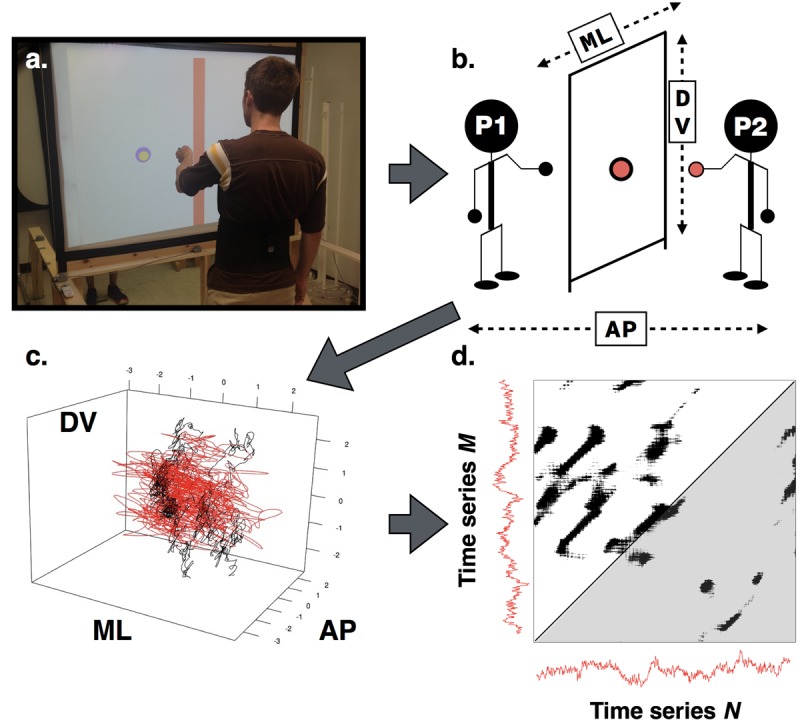
**(a)** View of the experimental set-up and apparatus. The performance meter is on the right of the screen. **(b)** Graphical depiction of participants engaged in the alignment task noting measurement axes. Each participant controlled a disk on the screen via a handheld motion sensor. As depicted here, Participant 1 controlled the larger disk (black), Participant 2 the smaller (gray), and both participants stood with their feet apart. To assess interpersonal coordination, participants’ movement time series **(c)** were submitted to cross-recurrence quantification analysis (CRQA). An example of a resulting cross-recurrence plot **(d)** where the lower gray shaded triangle indicates moments in time where time series M leads time series N, and the white shaded triangle indicates where N leads M.

We used a short throw projector to display a computer-generated scene onto a vertical white screen. The screen was translucent so that the projected scene could be seen on both sides. Pairs of participants stood facing one another on opposite sides of the screen (see **Figure 1**). Each participant stood approximately 1.2 m away from the screen.

In addition, we used a wireless 6DoF magnetic motion tracking system (Liberty Latus; Polhemus LTD, Colchester, VT, USA) to capture the position and orientation of participants’ body segments in 3-dimensional space. Each participant held one motion sensor in their dominant hand while another sensor was attached to their waist—providing densely sampled (94 Hz) data about the hand and torso movements. The position of each participant’s handheld sensor was mapped to a computer-generated avatar, a uniquely colored disk in the virtual scene constructed using custom software. By moving their hands, participants moved their respective disks—a displacement of the hand in the medial-lateral (ML) and superior-inferior (SI) axes resulted in an equal displacement of the disk on the screen [anterior-posterior (AP) hand movements did not affect the display]. As participants stood on opposite sides of the screen they could not see one another, but only the positions of one another’s disks. The projected disks were two sizes: 5 cm diameter and 8 cm in diameter, and the alignment task required that the participant with the smaller disk maintain their position within the perimeter of the larger disk. We selected these relative disk sizes as they demanded that participants precisely coordinate their movements to be successful in the alignment task, but allowed for enough flexibility that the task was not extraordinarily difficult (piloting suggested a less than 5% error rate for these sizes). The relative size of each participant’s disk was counterbalanced across trials.

### Procedure

During each trial, participants were asked to align their disks such that the smaller disk stayed within the perimeter of the larger disk (as in **Figure 1**). On a given trial each participant stood either with their feet shoulder-width apart (Easy) or in tandem heel-to-toe stance that provided an additional challenge to maintaining upright stance (Hard). This resulted in four possible dyad stances (Participant 1’s stance – Participant 2’s stance) conditions: Easy-Easy, Easy-Hard, Hard-Easy, and Hard-Hard. Stance conditions were crossed with control of the larger disk. Condition-trials were presented in random order in two blocks, resulting in 16 total trials. Each trial lasted for 45 s. To reduce the likelihood of arm fatigue, inter-trail intervals were a minimum of 30 s, at which time the experimenter would ask both participants if they were ready to continue. Participants were granted additional time if they so requested. Typically, breaks between trials lasted no longer than 60 s.

Participants were given real-time feedback about performance—a red dot appeared in the center of each disk when participants were out of alignment. In addition, participants were provided feedback about their overall performance via a meter on the edge of the projection. This meter decreased anytime the two participants were not in alignment with one another at a rate of 5% reduction for each second of error. Participants were told that the bar represented a performance score and that they should strive to keep the bar as full as possible and not allow it to become completely empty.

### Movement Analyses

Movement time series were collected for the *x, y, z* positions of each of the four markers. Movements in *x, y, z* corresponded to movements along the ML axis, AP axis, and SI axis, respectively. Prior to analysis, these data were smoothed using a 10-Hz Butterworth filter. The first 4 s of each time series was truncated to remove transients (as the participants settled into the alignment task).

When analyzing hand movements, we initially focused on positions along the ML and SI axes (movements in AP had no effect on the position of the avatar disk). When analyzing torso movements, we focused on ML and AP (as participants stood the entire time, no appreciable changes were expected in SI). However, meaningful effects in hand and torso were only found in the ML axes, therefore, for all reported data we focus on our measures as they relate to movement in the ML axis.

### CRQA: Global Dynamics and Leader-Follower Analyses

Time series data were submitted to CRQA. CRQA is a non-linear modeling technique that captures patterns of coordination between two interacting time series by indexing instances of their co-visitation in a shared, multidimensional phase space (Zbilut et al., 1998; Marwan and Kurths, 2004; Coco and Dale, 2014; Fusaroli et al., 2014) (see **Figure 1c**). The time series may be from different body segments of a single actor as in intra-personal coordination, or from two actors as in inter-personal coordination. Most natural systems have preferred states that they (approximately) revisit in stretches of repeating behavioral patterns, or recurrences (Poincaré, 1890). When dealing with dual time series, cross-recurrences may be interpreted as instances when one series is visiting a state that was occupied by the other at a previous point in time. The resulting structure of these cross-recurrences reveals important information about the organization and coordination dynamics of the system(s) under observation.

Cross-recurrence quantification analysis begins with the identification of cross-recurrent points and proceeds with several other measures that describe their relative number, density, distribution, and structure (Shockley, 2005). Visualizing these characteristics in reconstructed phase space is difficult when it has more than three dimensions. To this end, a simplified method involves indexing the cross-recurrent points between the embedded time series in a N × M binary matrix where N is the first time series and M is the second. Each point N_i_ that is determined cross recurrent with M_j_ is denoted with a mark at (i, j). CRQA is a quantitative analysis of this cross-recurrence plot (Eckmann et al., 1987; Marwan and Kurths, 2004) (see **Figure 1d**), and includes measures that highlight the density of cross-recurrent points, as well as their deterministic structure. For instance, the recurrence rate (RR) is the ratio of cross-recurrent points to all points in the phase space. RR is often used as an index of global coordination between two systems. When conducting CRQA, a non-trivial matter is the selection of the appropriate delay, embedding dimension, and radius parameters for the reconstructed phase space. Here, we selected the appropriate parameters for each trial based upon an optimization routine (Coco and Dale, 2014) using the average mutual information (Fraser and Swinney, 1986) and false nearest neighbors (Kennel et al., 1992) methods. The optimal radius was selected based upon the criterion that the final RR was between 3 and 5% (Shockley, 2005).

Successive or adjacent recurrent points form lines that reflect the structure of the coordination between the time series. Diagonal lines mark instances were the two series are co-evolving or moving parallel with one another through phase space. DET, or determinism, is a measure of the percentage of cross-recurrent points that form these diagonal line structures. Assuming a relatively constant RR, greater DET suggests stronger (i.e., more frequent) coupling between the time series. To assess changes in the complexity of coordination, we used a measure related to the Shannon information entropy of the diagonal line lengths in the recurrence plot. The Shannon information entropy is sensitive to the number of lines in the recurrence plot. Relative entropy (rENTR) accounts for this bias by normalizing the entropy value against the number of lines in the recurrence plot (Coco and Dale, 2014). This allowed us to more faithfully compare across trials and conditions.

The percentage of recurrent points forming vertical lines (*laminarity* or LAM), as well as the average vertical line length (*trapping time* or TT) index the proportion and average duration of laminar states. In auto-recurrence (when a time series is compared against itself), these vertical line measures are typically interpreted as capturing the degree of intermittency or rigidity (“stickiness”) in a system—that is how often and how long a system gets stuck in one or more states for a given behavior (Kiefer and Myer, 2015). When considered in the context of overt behavior, an actor that can smoothly and efficiently transition between and among stable states of behavior would exhibit lower rigidity values than an actor that does not transition effectively. Indeed, decreases in both LAM and TT have been associated with greater functional flexibility in skill acquisition or development (Wallot and Grabowski, 2013; Kiefer and Myer, 2015). However, in cross-recurrence (Cox and van Dijk, 2013), these measures take on a slightly different meaning. In the context of two actors, M and N (see **Figure 1d**), the vertical line measures speak to actor M visiting a single point in phase space, and then actor N visiting that same space over consecutive temporal samples even as actor M has moved on. This could indicate that actor M led or constrained actor N into a certain movement pattern, before moving on, with the result that actor N maintains or is stuck in that movement pattern for a certain length of time longer than the duration of time actor M spent in that same space, or trajectory.

Leader-follower relationships may be further assessed by taking note of the symmetry properties of the cross-recurrence plot. In the cross-recurrence plot, an imaginary line of incidence (LOI) runs along the diagonal where N_i_ = M_j_. This line represents points where both time series are exhibiting a 0-lag synchronization over consecutive samples. Cross-recurrent points in the triangular regions above and below the LOI represent points in time when one time series is revisiting a state previously occupied by the other at a given time delay (i.e., >0 lag in either direction). For example, cross-recurrent points where N_i_ > M_j_ indicate that point N is visiting a state previously occupied by M and cross-recurrent points where N_i_ < M_j_ indicate the opposite. While CRQA measures regarding the entire cross-recurrence plot provide metrics of the global dynamics, evaluating these regions separately allowed us to compare the structure of coordination as a consequence of which time series was ahead of the other. For example, greater DET in the upper region compared to the lower region in **Figure 1d** would suggest that the coordination between the two series is more tightly coupled when time series M is entering states at consecutive time points previously occupied by time series N over consecutive time points, but with a time-lag greater than 0.

Time lags are able to be quantified via the orthogonal distance from any cross-recurrent point to the LOI. A measure of the diagonal-wise RR, then, provides a measure of the density of recurrent points at a particular time lag (a measure analogous to cross-correlation). A simple measure of diagonal-wise RR involves indexing the lag at which it is greatest within a selected window around the LOI, providing a measure of the degree of leader-follower relationships between the time series (Dale and Spivey, 2006; Warlaumont et al., 2014). Here, we refer to this value as LAGMAX.

### Measures and Design

Intra-personal analysis included measures of movement variability of hand and torso, as well as CRQA measures of within-individual hand-torso coordination. Inter-personal analyses focused on the coordination between the two actors’ hands, including both CRQA measures and task performance measures. For both levels of analyses, we tested for differences in actors’ behavior as a function of their relative stances (dyad stance: Easy-Easy, Easy-Hard, Hard-Easy, and Hard-Hard) and which actor controlled the larger disk. When one actor controlled the smaller disk, the other, by definition, controlled the larger disk. We considered that this manipulation may have defined distinct, *a priori* roles for the dyad members—for example the larger disk may be interpreted as a boundary for the smaller disk to remain within.

Both intra-personal and inter-personal analyses included a 2 (disk control) × 4 (dyad stance) repeated measures design. In the case of intra-personal coordination, we were also concerned with identifying differences between co-actors. As such, in our intra-personal analyses we crossed disk control and dyad stance with a between-factor for actor (Person 1 or Person 2 of the dyad). In the case of inter-personal coordination, our concern was not in differences between actors, *per se*, but instead differences in coordination as a function of which actor was moving ahead of, or leading, the other. Therefore, for these analyses, disk control and dyad stance were instead crossed with an additional within-factor to account for differences in CRQA measures as a function of triangular region (upper triangle or lower triangle).

## Results

### Individual Level Analyses

#### Movement Variability

We quantified movement variability as the standard deviation of effector (hand and torso) position during each trial. While hand movement variability tended to be greater in the Hard-Hard dyad stance relative to the Easy-Easy dyad stance, no significant main effects nor any interactions were observed for either hand or torso (*ps* > 0.05).

### Hand-Torso Coordination

Analysis of variance (ANOVA) of intra-personal hand-torso DET revealed a dyad stance × actor interaction [*F*(3,66) = 16.92, *p* < 0.001, $\eta_{p}^{2}$ = 0.43]. When dyads were in identical stances (Easy-Easy and Hard-Hard) there was no difference in DET between actors. When actors were in different stances (Easy-Hard and Hard-Easy) the actor in the Hard stance condition exhibited greater DET between hand and torso. Overall, individuals’ DET was greater when actors were in the Hard stance compared to the Easy stance. ANOVA also revealed a dyad stance × control interaction [*F*(3,66) = 5.84, *p* = 0.001, $\eta_{p}^{2}$ = 0.21].

**FIGURE 2:**
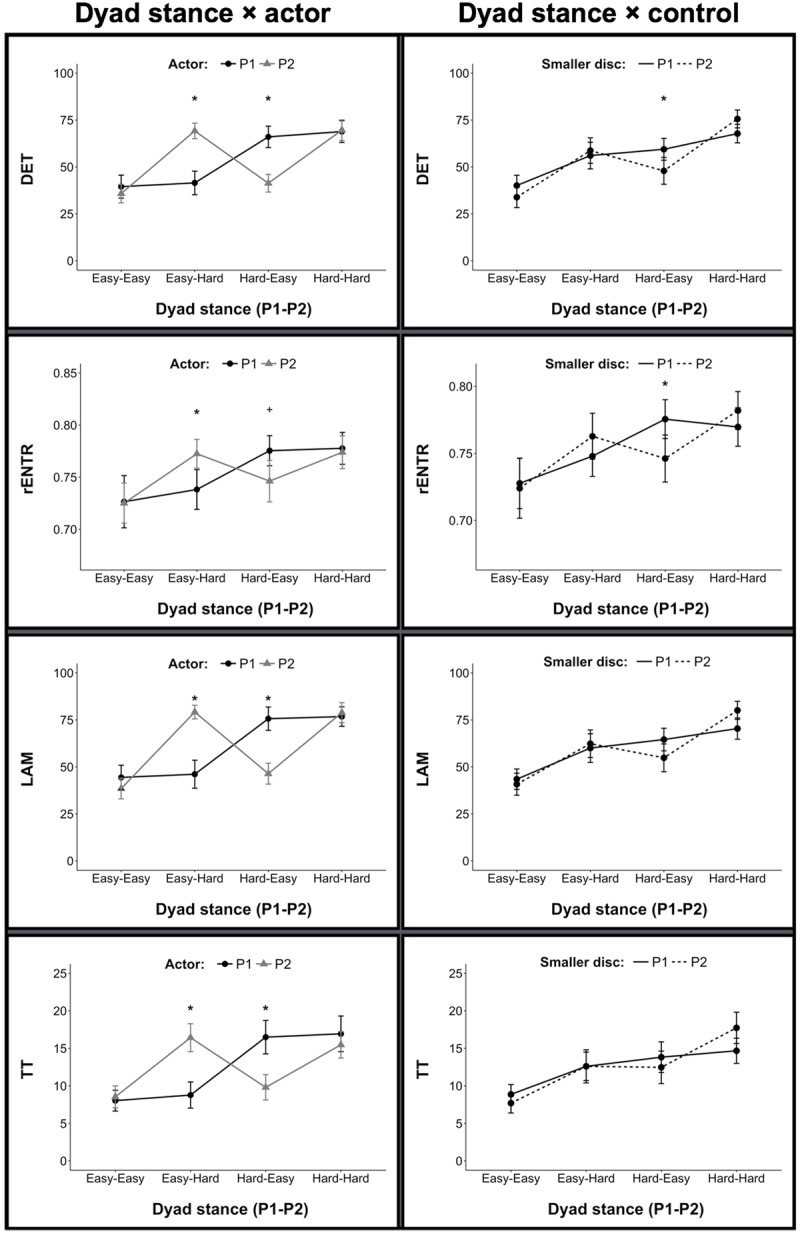
**Measures of intra-personal hand-torso coordination.** Here and in all remaining figures (unless otherwise noted), error bars represent 95% confidence intervals. (^∗^) denotes *p* < 0.05; (+) denotes *p* < 0.10.

Similar patterns of significant effects (see **Table 1**) were observed for rENTR, LAM, and TT, however, we note that the dyad stance × control interaction was non-significant (*p* = 0.060) for TT. A graphical representation of these effects may be found in **Figure 2**.

**Table 1:** Intra-personal coordination effects.

Effect	DET	rENTR	LAM	TT
Dyad stance	*F*_(3,66)_ = 24.39^∗∗^	*F*_(3,66)_ = 13.25^∗∗^	*F*_(3,66)_ = 25.89^∗∗^	*F*_(3,66)_ = 29.57^∗∗^
Dyad stance × actor	*F*_(3,66)_ = 16.92^∗∗^	*F*_(3,66)_ = 4.75^∗^	*F*_(3,66)_ = 19.21^∗∗^	*F*_(3,66)_ = 17.30^∗∗^
Dyad stance × control of smaller	*F*_(3,66)_ = 5.84^∗^	*F*_(3,66)_ = 3.30^+^	*F*_(3,66)_ = 3.91^+^	*F*_(3,66)_ = 3.08 (*ns*)


^+^*p < 0.05; ^∗^p < 0.01; ^∗∗^p < 0.001.*

#### Summary of Individual-Level Analyses

Before continuing to our inter-personal data, we briefly revisit the intra-personal results and their implications. First, we hypothesized that our stance manipulation would impact actors’ postural stability. However, our initial analysis of the data did not yield the anticipated increases in torso sway variability. In light of previous conflicting findings (Ramenzoni et al., 2011), we considered the possibility that any individual changes in movement variability may have been obscured by the design of our original analysis. For example, Ramenzoni et al. (2011) analyzed movement variability as a function of each actor’s own stance (e.g., Easy stance) independent of the stance of their partner (e.g., Hard stance); while our primary concern was how each actor’s demands related to their partner’s provided a particular stance (e.g., dyad stance: actor in Easy and partner in Hard). To address this possibility, we re-analyzed each actor’s hand movement and torso movement variability only as a function their own stance (Easy or Hard) and their prescribed control (larger or smaller disk). While torso movement variability tended to be greater when actors were in the Hard stance (*p* = 0.058), hand movement variability remained indifferent to these two factors. Indeed, it has been observed that variability in goal-directed arm movements may remain immune to the effects of increased postural challenges (Voudouris et al., 2013). Here, the observation that hand movement variability was relatively immune to the independent effects of one’s own stance suggests that individuals’ hands and torsos were behaving in a synergistic fashion to meet the precision demands of the task.

Our data offers additional support for the intra-personal synergy hypothesis—actors in our task reorganized the coordination between their hand and torso to compensate for increased challenges to stance. More specifically, individual actors exhibited greater regularity (DET) and complexity (rENTR) of intra-personal coordination when in the more difficult stance condition. These changes were accompanied by increased intermittency (increases in LAM and TT) in the coordination between hand and torso. Moreover, consistent with our hypotheses, when pairs of actors faced different stance demands, these measures differentiated pairs of actors in mixed-stance conditions (Easy-Hard and Hard-Easy); but were similar across actors when they performed the task while in identical stances (Easy-Easy and Hard-Hard).

Taken together, these intra-personal results had important implications for our inter-personal analyses. First, they supported the broad hypothesis that our actors faced differing task demands due to our experimental manipulations. These differences resulted in systematic changes in patterns of intra-personal coordination between hand and torso. We further hypothesized that differences in intra-personal constraints and coordination would result in differences in the observed patterns of inter-personal coordination—most notably the leader-follower relationship between members of the dyad.

For example, one interpretation of the LJH framework would predict that that the actor exhibiting greater complexity in intra-personal coordination should be the follower in the joint task. In our present study, complexity was indexed by the relative entropy of cross-recurrences between hand and torso (rENTR). Given our intra-personal results, this hypothesis would predict that when actors were in mixed stances, the actor in the more difficult stance (greater rENTR) would be more likely to be the follower in coordinating to meet the joint precision task. Conversely, in the same stance conditions no differences were observed between actors’ intra-personal rENTR suggesting that leader-follower relationships in these conditions were less like to be systematic. In what follows, we test this hypothesis as it relates to our data.

### Dyad Level Analyses

#### Leader-Follower Analyses

**FIGURE 3:**
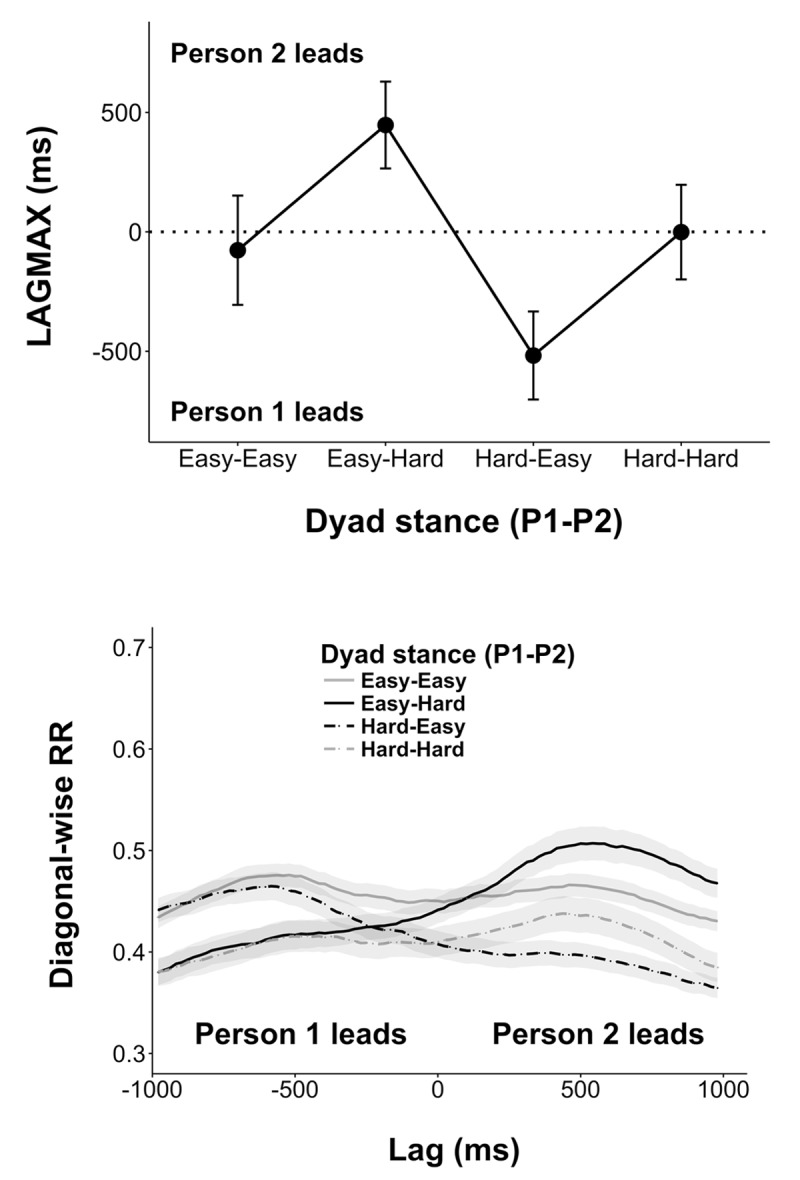
**(Top)** LAGMAX as a function of dyad stance. Lags less than zero indicate that Person 1 lead the coordination at the hands; greater than zero indicates Person 2 led. As illustrated here, a leader-follower dynamic emerged in conditions were actors were in mixed-stance conditions, where the actor in the Hard stance tended to lead the actor in the Easy stance. **(Bottom)** Mean lag profiles (±1000 ms) of each stance condition. Mixed dyad stance conditions (Easy-Hard and Hard-Easy) are in black. Note that these conditions produce greater asymmetries in diagRR about 0 compared to the relatively flat curves in conditions when actors were in similar stances (in gray). Error bars represent standard error.

When considering inter-personal coordination, LAGMAX indexed the degree to which one of the participants led the other in coordinated movement. Here, positive LAGMAX indicated that Person 2 led the coordination, and negative LAGMAX indicated that Person 1 led. ANOVA confirmed that dyad stance had a significant effect on inter-personal LAGMAX for hands [*F*(3,33) = 26.02, *p* < 0.001, $\eta_{p}^{2}$ = 0.70]. As we were concerned with whether this manipulation produced a meaningful lead-lag, we tested whether each resulting condition mean was different from zero by using 95% confidence intervals where 0 ∉ Mean ± 95% CI was considered significant. Consistent with our predictions of leader-follower emergence, mean LAGMAX was significantly greater than zero in the Easy-Hard (Person 1-Person 2) stance condition (447 ± 182 ms) and less than zero in Hard-Easy condition (-517 ± 184 ms). When actors were in identical stances there was no significant lag (see **Figure 3**). No statistically significant effect was observed for disk control nor was there any observed interaction (*ps* > 0.05).

#### Interpersonal Hand Coordination

**FIGURE 4:**
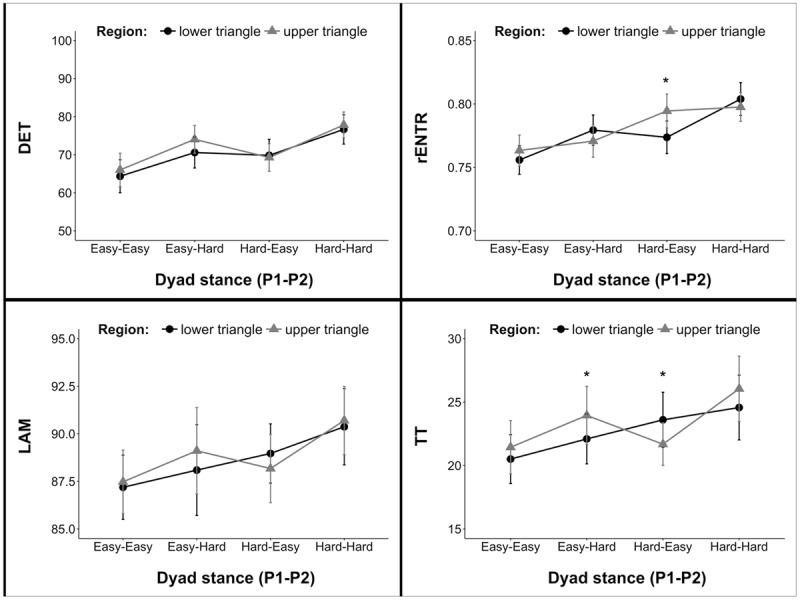
**Inter-personal hand coordination measures as a function of dyad stance and region.** In general, measures were lowest in the Easy-Easy stance and greatest in the Hard-Hard stance. Mixed stance (Easy-Hard and Hard-Easy) were typically intermediate. Note that measures of TT in mixed-stance conditions differed by region–indicating greater TT during moments in time when the actor in Hard stance lead the coordination.

Main effects of dyad stance were found for DET [*F*(3,33) = 9.04, *p* < 0.001, $\eta_{p}^{2}$ = 0.45], rENTR [*F*(3,33) = 10.26, *p* < 0.001, $\eta_{p}^{2}$ = 0.48], LAM [*F*(3,33) = 3.72, *p* < 0.021, $\eta_{p}^{2}$ = 0.25], and TT [*F*(3,33) = 4.37, *p* < 0.011, $\eta_{p}^{2}$ = 0.28]. Each measure increased as a function of dyad stance difficulty: Easy-Easy was lowest, Easy-Hard and Hard-Easy were intermediary, and Hard-Hard was highest (see **Figure 4**). Moreover, dyad stance × triangle interactions were observed for TT [*F*(3,33) = 3.18, *p* < 0.037, $\eta_{p}^{2}$ = 0.22] and rENTR [*F*(3,33) = 5.43, *p* = 0.004, $\eta_{p}^{2}$ = 0.33]. In the asymmetrical dyad stance conditions, TT was greater in periods when the time series of the actor in the Hard stance was ahead of the actor in the Easy stance. No significant differences were observed when actors were in identical stances. The interaction effect for rENTR was primarily driven by a simple effect for triangle in the Hard-Easy stance condition. No simple effects for triangle were observed in the remaining dyad stance conditions.

Notably, disk control did not have a significant main effect on any of our output measures.

#### Task Performance

We measured task performance on each trial in two manners. An overall score was provided by the height of the performance meter, which was in turn a function of the amount of time spent successfully performing the alignment task. In addition, a continuous time series of inter-disk distances was used to analyze the precision with which participants performed the task.

Neither dyad stance nor control had any significant effect on overall task performance (the amount of time spent in alignment). However, dyad stance did have an effect on the average distance between the center of the two actors’ disks [*F*(3,33) = 3.53, *p* = 0.025, $\eta_{p}^{2}$ = 0.24]. Overall, when participants were in the Easy-Easy stance condition, they kept their avatar disks in tighter alignment (mean distance: 0.69 cm; *SD*: 0.12 cm) compared to the remaining three stance conditions (mean distances all greater than 0.75 cm). That said, participants were able to perform the task exceptionally well in all conditions and overall task performance was preserved in spite of increases in stance difficulty.

#### Summary of Joint-Level Analyses

Consistent with previous work (Ramenzoni et al., 2011) we found our measures of inter-personal coordination varied as a function of our dyads’ shared stances. The regularity (DET) and complexity (rENTR) of coordination between actors’ hands increased from Easy-Easy to mixed stance (Easy-Hard and Hard-Easy) to Hard-Hard stance conditions. Analogous increases in our laminar measures (LAM and TT) indicate that flexibility decreased in a commensurate manner. Put another way, these changes tracked with the increases in the combined stance difficulty—when both actors were in the Easy stance their combined stance difficulty was relatively lower than the challenges faced when both actors were in the Hard stance, while mixed conditions were intermediary. Viewed in this light, the pattern of inter-personal coordination effects is consistent with our observed intra-personal effects. When faced with additional challenges to completing the task, actors compensated in similar manners at both levels of coordination.

Importantly, as indicated by observed LAGMAX data, we found evidence of leader-follower dynamics between actors. These observations were consistent with our general working hypothesis—leader-follower relationships in inter-personal hand coordination were most pronounced in conditions when actors faced asymmetric stance demands. This result was supported by TT measures indicating that in mixed-stance conditions the average duration in which one actor was stuck in a state previously occupied by another was greater in regions where the actor in Hard stance entered those states first. Notably, the direction of this relationship was not as we predicted given observed differences in the complexity of intra-personal coordination. Motivated by findings that extend the LJH to joint action, we predicted that actors exhibiting greater complexity in intra-personal coordination would be more likely to follow in the joint task. However, our results indicate the opposite—actors in the Hard stance, though typically exhibiting greater intra-personal rENTR, tended to be leaders in the interpersonal hand coordination.

### Analysis by Role

#### Motivation and Model Definition

Our results indicated the emergence of leader-follower roles in interpersonal hand coordination was most pronounced in conditions were co-actors faced asymmetrical stance demands. In light of this result we re-analyzed the intra-personal dependent measures as a function of each actor’s role (leading vs. following), actor’s control (smaller disk vs. larger disk) and the dyad’s stance symmetry (different stances vs. same stances). A leader and a follower was determined for each trial using the inter-personal hand LAGMAX values. Because participants were not experimentally assigned to “role” and, therefore, our groups were unbalanced, we determined the relationship between our dependent measures and factors using a linear mixed effects regression model with emergent role, actor’s control, and stance demands as fixed effects and dyad as a random effect. For brevity we present only the *F*-tests from the results here (type III Wald *F*-tests with Kenward–Roger degrees of freedom approximation).

#### Intra-Personal Coordination as a Function of Leader-Follower

**FIGURE 5:**
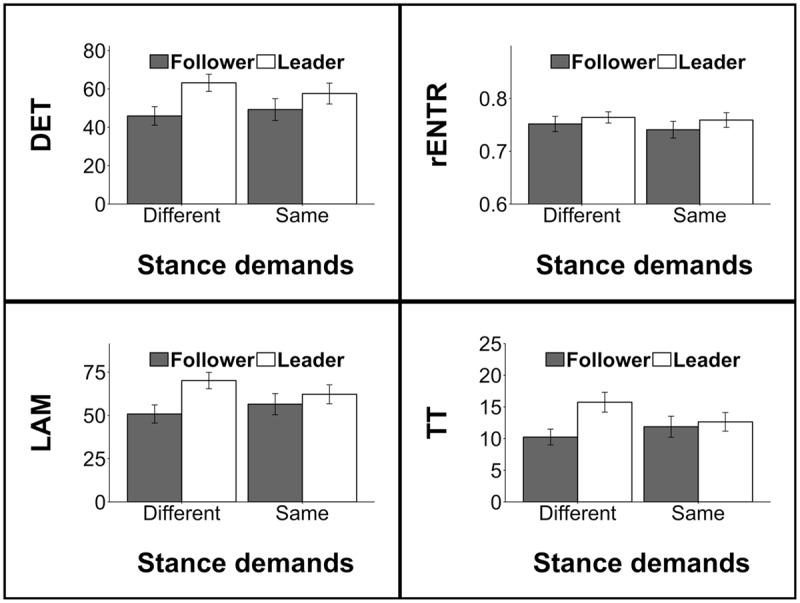
**Intra-personal coordination measures for *leaders* and *followers*.** Differences in measures were exaggerated in trials were actors were in non-identical stances.

As we anticipated, intra-personal coordination measures were observed to vary along with emergent role. Overall, leaders exhibited stronger hand-torso coupling (DET) than followers [*F*(1,25.3) = 15.67, *p* < 0.001]. This difference was exaggerated when actors faced different stance demands [interaction effect: *F*(1,206.7) = 7.22, *p* = 0.007]. Similar relationships were also observed for LAM [leaders greater than followers: *F*(1,26.5) = 18.28, *p* < 0.001; interaction effect: *F*(1,207.3) = 10.83, *p* = 0.001] and TT [leaders greater than followers: *F*(1,26.5) = 12.54, *p* = 0.002; interaction effect: *F*(1,202.0) = 12.52, *p* < 0.001]. Leaders also exhibited greater rENTR than followers [*F*(1,23.3) = 6.13, *p* = 0.021], however, no interaction was observed (see **Figure 5**). Notably, similar linear effects mixed regression models for hand movement and torso movement variability did not reveal significant results—neither varied according to emergent role.

## General Discussion

When two people organize their actions to achieve a shared goal, their combined efforts reflect a nested structure of intra-personal and inter-personal coordination. Combined efforts, however, almost never equate to identical efforts. Differences between actors’ skill and physical abilities often result in asymmetries in task demands and, as a result, individuals working together often need to perform distinct and complementary actions in order to complete a shared task. In the present study, we investigated how these asymmetries influence both intra-personal and inter-personal coordination during a joint supra-postural task, focusing on the spontaneous emergence of leader-follower roles when performing this cooperative task.

To briefly revisit our hypotheses, on the outset we predicted that (1) actors in the tandem stance would face greater individual challenges to postural stability (as indexed by movement variability) compared to actors in the feet-apart stance, and (2) these increases in stance difficulty would reflect in the coordination between hand and torso to meet the task’s precision demands. More specifically, we anticipated that (3) actors in the difficult, tandem stance condition would exhibit greater complexity and intermittency in intra-personal coordination compared to actors in the easier feet-apart condition. Provided this result, we predicted that (4) actors in the Hard stance condition would be more likely to follow their Easy-stance confederates—that is, their hand movements would slightly lag behind the movements of partners in the easier stance. We predicted that (5) these lead-lag relationships would be most systematic during trials when actors were in different stances. When actors were in identical stances we anticipated that the presence of lead-lag would be less pronounced.

Our data support many of our original hypotheses, with a caveat regarding hypothesis 1 and an exception for hypothesis 4. At the level of individuals, we found evidence that actors in tandem faced additional challenges during the joint task. While our analyses revealed no changes in hand movement variability, they hinted at increased postural sway variability for actors in tandem stance. More notably, actors in tandem stance did exhibit changes in the organization of intra-personal coordination in line with our prediction and consistent with previous literature. These changes included increases in the regularity, intermittency, and complexity of coordination between hand and torso in order to meet the precision task demands. One interpretation for this result is that actors in the more difficult stance condition faced a reduction in the number of available states (degrees of freedom) that they could occupy, or were willing to occupy, and still complete their task. For example, when in the tandem stance, actor’s movements needed to be more tightly constrained lest the actor lose their balance.

When framed as above it is perhaps not surprising that—contrary to our predicted direction— actors in the more difficult stance tended to lead their Easy-stance confederates. Actors with compromised postural stability may have had less opportunity or flexibility to adapt to the activity of their partners. In turn, the more stable and meta-flexible actor—the actor who was able to respond to their partner by optimizing their rigidity and flexibility without becoming stuck or falling apart (Pincus and Metten, 2010)—use their flexibility for the benefit of the dyad (indeed, analogs to these sorts of counterbalancing relations abound in the motor literature with respect to injury and compensatory reorganization of other body segments—much like when one’s right leg bears an additional load if the knee or ankle of the left legs is sprained). Our dyads were able to organize their actions to meet the shared task demands—the challenges and changes had no appreciable effect of the degree to which pairs of individuals were able to complete the task. Thus, while individuals were able to work together with similar competence across our experimental conditions, they organized their intra-personal and inter-personal activity in very different manners depending on the prevailing task constraints.

It is also worth noting potential distinctions between the complexity measures used here and those often employed in research regarding the LJH. In particular, the LJH predicts that the increased variability and complexity about the subordinate joints is the result of the subordinate joints resolving interacting torques that are produced during action. As such, the hypothesis makes specific claims about components that are mechanically linked. This was also the case in Bosga et al.’s (2010) extension to joint action—actors movements were mechanically linked via a rocking board. Here, no such linkages were present between actors. Instead our actors were informationally linked. Though informational couplings have been shown to produce constraints similar in kind with mechanical couplings, it is routinely the case that there are important differences in the characteristics of the coupling produced (Schmidt and Richardson, 2008). Our results suggest that the LJH may not be the appropriate framework for addressing tasks of these sorts. At the same time, the rENTR measure may not be synonymous with the complexity measures typically employed in the LJH literature. Rather than focus on movement fluctuations between body segments rENTR speaks to the complexity/homogeneity of their coupling through time.

Our central focus was identifying relationships between individual task demands, individual task dynamics, and the self-organization of leader-follower roles in joint tasks. To this end our data demonstrate systematic relationships between individual task difficulty, the organization of action within individuals (intra-personal coordination) and the organization of action across individuals (inter-personal coordination). It is notable that the pattern of effects for both intra- and inter-personal coordination were similar in kind. Measures of inter-personal hand coordination tended to increase as a function of the dyad’s shared-stance difficulty—lowest when both members of the dyad were in an Easy stance, greatest when both members were in the Hard stance, and intermediate when the individual stance difficulties were mixed. These results suggest similar compensatory processes occurring within and across individuals in order to meet changes in individual and joint task demands. Whether these increases were a functional response to the task demands or a result of a reduction in available degrees of freedom remains an open question.

Interestingly, the *a priori* assignment of role (disk size) appeared to have no appreciable effect on emergent role—that is, who controlled the larger disk or the smaller disk did not have any statistically significant influence on who led and who followed. We note, however, that using a similar paradigm, Davis et al. (2016), were able to identify a relationship between disk control and dyadic performance using a complementary non-linear analysis technique, multi-fractal detrended fluctuation analysis. That no effect was found here may be due to the lack of sensitivity of our CRQA measures, or lack of additional manipulations specifically targeting this factor.

Most germane to our study, we observed that members of the dyad organized their activity into leader-follower roles when facing asymmetrical task demands. While lead-lag relationships have been investigated using CRQA methods in conversational settings (Richardson and Dale, 2005; Dale and Warlaumont, 2011) only recently have similar methods been directed at the lead-lag analysis of body movements during goal-directed joint activities (Abney et al., 2015). Here we employed an analysis that allowed us to further compare the deterministic structure of inter-personal coordination depending upon when one actor “took the lead” compared to the other. In particular, our results regarding the laminar states of intra-personal and inter-personal coordination are revealing. Increases in LAM and TT in intra-personal coordination suggest that actors in more difficult stance conditions may have had more difficulty (or reluctance) transitioning among available stable states of behavior. Scaling up to the level of the dyad, inter-personal coordination measures indicated a lack of flexibility in the coordination between the pair, with one visiting and becoming trapped in a state that the other had previously occupied. In this regard, the multi-agent coordination was driven based on the relative flexibility of each of the actors in achieving their individual task demands. Thus, it may be possible in the future to assess the performance weight of each component of a coupled system as an indicator of who would lead and who would follow in the group, with overall group performance indexed via the laminarity measure of the dyad.

Our results, by extension, also suggest that whatever dynamics are observed at larger scales may also be observed (although, perhaps not always manifestly apparent) at smaller scales within multi-agent activity. When considering interpersonal synergies, the character of the synergy should be identifiable at any insertion point of measurement—we may characterize the collective behavior of a multi-agent system through measurement of overall group dynamics. While such an approach may not allow for the direct comparison between all members, it may provide a level of prediction that would suffice for probabilistic behavior (or behavioral capacities) of the group. More broadly, this result is consonant with recent efforts to address interpersonal activity within the framework of *interaction*-*dominant dynamics* (Van Orden et al., 2003; Diniz et al., 2011; see, for example, Riley et al., 2011). In contrast to component dominant dynamics, which characterizes systems in terms of local-scale effects between relatively static structures, interaction-dominant dynamics are characterized by effects across a range of scales. The observed similarities between intra- and inter-personal coordination dynamics do not by themselves provide conclusive evidence that the dyadic coordination in the present task represents an interaction-dominant system. Indeed, our analysis does not directly test for this possibility. However, when couched with recent investigations of interpersonal coordination that more explicitly address this possibility (e.g., Bedia et al., 2014; Dumas et al., 2014)—including in a similar task (Davis et al., 2016)—our finding bolsters this hypothesis. An important takeaway from these results, then, is that a proper characterization of interpersonal behavior may necessitate looking across scales, as it is likely that multiple scales of activity are contributing to the global dynamic.

## Conclusion

To successfully engage in a joint action, individual actors must often resolve their own, local task demands. How individuals meet these demands may, at times, be wholly intrinsic, but more than likely is due to the influence of the activity of other co-actors. Here, we showed how individual task demands influenced coordination at the intra-personal and inter-personal scales, most prominently resulting in the organization of leader-follower roles in the joint action. Given that our actors did not have any specific knowledge of one another’s task demands, this raises the possibility that the observed activity was organized around some informational variable related to the visual display—that is, there may have been something in the way the disks moved that influenced the emergence of roles in the present task. Future directions may seek to explore this possibility, and may offer further avenues of inquiry in the relationships between individuals and groups in joint actions.

## Ethics Statement

All procedures were conducted in agreement and accordance with the guidelines and approval of the University of Connecticut Institutional Review Board. Each participant provided verbal informed consent prior to the start the study.

## Author Contributions

TD conceived of the study, designed the experiments, and drafted the manuscript. GP collected the data and performed the CRQA and statistical analyses. AK drafted the manuscript and made important theoretical contributions. All authors have read and approved the final version of the manuscript, and agree with the order and presentation of the authors.

## Conflict of Interest Statement

The authors declare that the research was conducted in the absence of any commercial or financial relationships that could be construed as a potential conflict of interest.
